# Genome-wide identification of expression quantitative trait loci for human telomerase

**DOI:** 10.1097/MD.0000000000005209

**Published:** 2016-10-21

**Authors:** Hanseol Kim, Jihye Ryu, Chaeyoung Lee

**Affiliations:** Department of Bioinformatics and Life Science, Soongsil University, Seoul, Korea.

**Keywords:** expression quantitative trait locus, genome-wide association study, single nucleotide variant, telomerase, Wnt signaling pathway

## Abstract

A genome-wide association study was conducted to identify expression quantitative trait loci (eQTL) for human telomerase.

We tested the genetic associations of nucleotide variants with expression of the genes encoding human telomerase reverse transcriptase (hTERT) and telomerase RNA components (TERC) in lymphoblastoid cell lines derived from 373 Europeans.

Our results revealed 6 eQTLs associated with hTERT (*P* < 5 × 10^–8^). One eQTL (rs17755753) was located in the intron 1 of the gene encoding R-spondin-3 (RSPO3), a well-known Wnt signaling regulator. Transcriptome-wide association analysis for these eQTLs revealed their additional associations with the expression of 29 genes (*P* < 4.75 × 10^–6^), including prickle planar cell polarity protein 2 (PRICKLE2) gene important for the Wnt signaling pathway. This concurs with previous studies in which significant expressional relationships between hTERT and some genes (β-catenin and Wnt-3a) in the Wnt signaling pathway have been observed.

This study suggested 6 novel eQTLs for hTERT and the association of hTERT with the Wnt signaling pathway. Further studies are needed to understand their underlying mechanisms to improve our understanding of the role of hTERT in cancer.

## Abstract 2

Supplemental Digital Content is available in the text

## 1 Introduction

Telomeres play key roles in human genome stability through replenishing their G-rich sequences by telomerases.^[1]^ A variety of human cancer cells show high expression levels of telomerase with short telomeres whereas most human somatic cells have low expression levels of telomerase.^[2]^ The telomerases consist of human telomerase reverse transcriptases (hTERT) that regulate telomerase activity and telomerase RNA components (TERC) that are used as template RNAs for lengthening telomeres.^[2]^

It has been reported that mRNA expression level of hTERT is highly correlated with telomerase activity in tumor cells.^[3]^ TERC are targeted by imetelstat sodium (GRN163L), an inhibitor of telomerase activity in cancer therapy.^[4]^ Nevertheless, genetic factors that regulate the variability of gene expression are not well understood. In particular, knowledge on genetic factors is limited to genetic variability in or near the genes encoding hTERT and TERC. Some nucleotide variants (rs2736108 upstream of hTERT, rs7705526 in intron 2 of hTERT, and rs12696304 downstream of TERC) were associated with telomere length in leukocytes.^[5,6]^ Some intronic variants (rs10069690, rs2242652, and rs7725218) of hTERT have been reported to have association with hTERT expression in prostate cancers.^[7]^ This study aimed to identify nucleotide variants associated with mRNA expression of hTERT and TERC through genome-wide analysis.

## 2 Material and methods

### 2.1 Subjects

Expression data of the genes encoding hTERT and TERC in lymphoblastoid cell lines generated from the Geuvadis RNA-sequencing project^[8]^ were used to identify expression quantitative trait loci (eQTLs). Cell lines were derived from 373 Europeans of the following 4 populations: Utah residents with northern and western ancestry (n = 91), Finns (n = 95), British (n = 94), and Toscani (n = 93).^[8]^ We excluded Yoruba population from the project to avoid false positive associations produced by heterogeneous genetic background. Gene expression was calculated as the sum of reads per kilobase per million mapped reads (RPKM) for all transcripts of each gene in each individual.^[8]^ Their corresponding genotypic data were obtained from the 1000 Genomes Project (http://www.1000genomes.org/). Genotypes with minor allele frequency <5% or with missing rate of >5% were removed. After the quality control, genotypes of 5,851,914 SNPs were used for final analysis. Ethical approval was not necessary because we dealt with publically available data.

### 2.2 Statistical methods

Linear regression analysis was performed to discover eQTLs of hTERT and TERC. Multiple testing was employed with significance threshold value of 5 × 10^–8^. All the statistical analyses were conducted using PLINK.^[9]^ Linkage disequilibrium (LD) blocks at association signals were constructed using HaploView.^[10]^ The identified eQTLs were analyzed to determine if they were located in transcription factor binding sites using ChIP-seq data from the regulomeDB.^[11]^

### 2.3 Transcriptome-wide association analysis of eQTLs for human telomerase reverse transcriptase

Further associations between 10,518 genes and SNPs identified to be associated with the expression of hTERT were examined. For transcriptome-wide association analysis, the significance threshold value was set at 4.75 × 10^–6^ (= 0.05 divided by the total number of 10,518 genes). Functional enrichment analysis was conducted with the identified genes to examine their functional relevance using the DAVID functional annotation tool.^[12]^

## 3 Results

**Figure 1:**
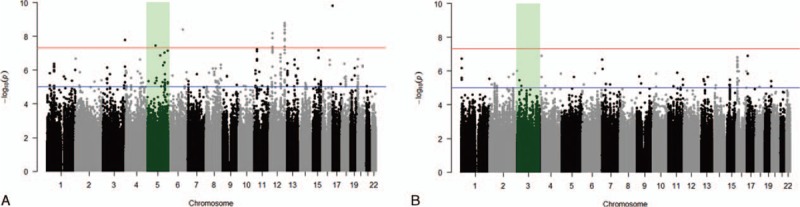
Manhattan plot for genome-wide association between eQTLs and mRNA expression of hTERT (A) or TERC (B). The red line indicates a genome-wide significance threshold (*P* = 5 × 10^–8^), and the blue line indicates a threshold for “suggestive” variants (*P* = 10^–5^). The shadow indicates cis regulation. eQTLs = expression quantitative trait loci, hTERT = human telomerase reverse transcriptase, TERC = telomerase RNA components.

**Figure 2:**
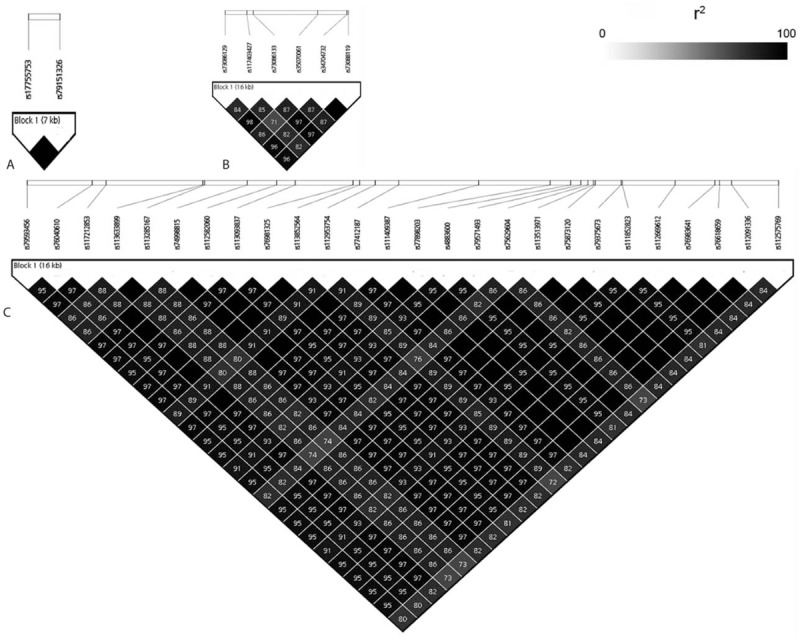
Linkage disequilibrium blocks for signals identified to have association with the expression of hTERT. (A) 6:127,145,296–127,153,155; (B) 12: 25,867,980–25,884,637; (C) 12:132,426,458–132,443,036. hTERT = human telomerase reverse transcriptase.

Genome-wide association analysis revealed 37 eQTLs associated with mRNA expression of hTERT (*P* < 5 × 10^–8^; Fig. 1, Table 1). However, no significant eQTL was identified to be associated with mRNA expression of TERC (*P* > 5 × 10^–8^; Fig. 1). Some of the identified eQTLs located in chromosomes 6 and 12 turned out to be in strong linkage, and linkage disequilibrium blocks were constructed in Fig. 2. As a result, we found 6 association signals (rs224514, rs112953754, rs17755753, rs35070061, rs2636908, and rs187444335) from the eQTL analysis (Table 1). Among them, the intronic nucleotide variant rs224514 of the gene encoding sedoheptulokinase (SHPK) showed the most significant association signal (*P* = 1.50 × 10^–10^). Another intragenic association signal was located in intron 1 of R-spondin-3 (RSPO3) gene (rs17755753, *P* = 3.91 × 10^–9^).

**Table 1:**
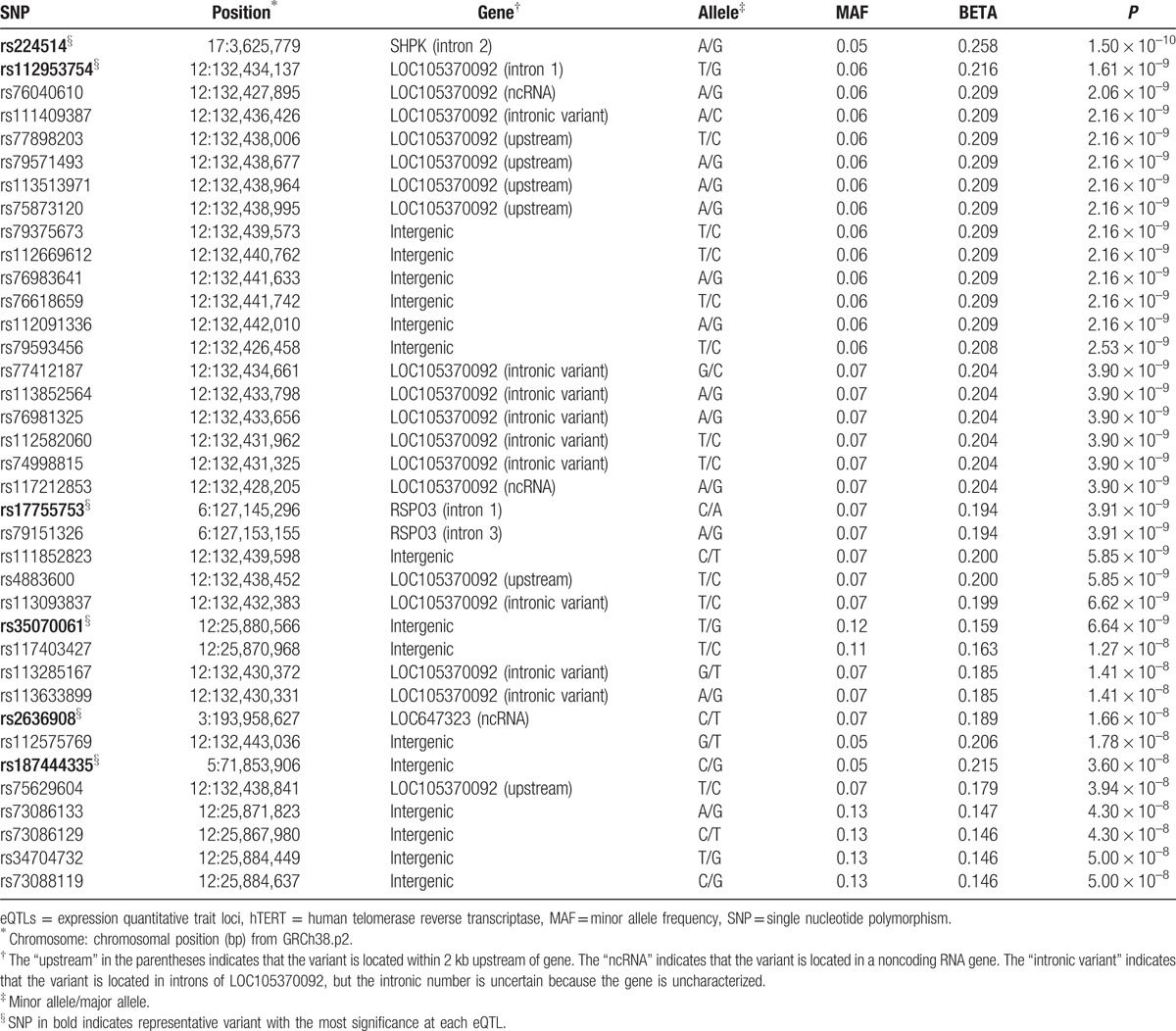
Genome-wide associations of SNPs with mRNA expression of hTERT gene.

Transcriptome-wide association analysis with the 6 eQTLs identified to have association with hTERT expression showed significant associations with mRNA expressions of additional 29 genes (*P* < 4.75 × 10^–6^; Table 2). No gene other than hTERT was identified to have significant association with rs35070061 (*P* > 4.75 × 10^–6^).

**Table 2:**
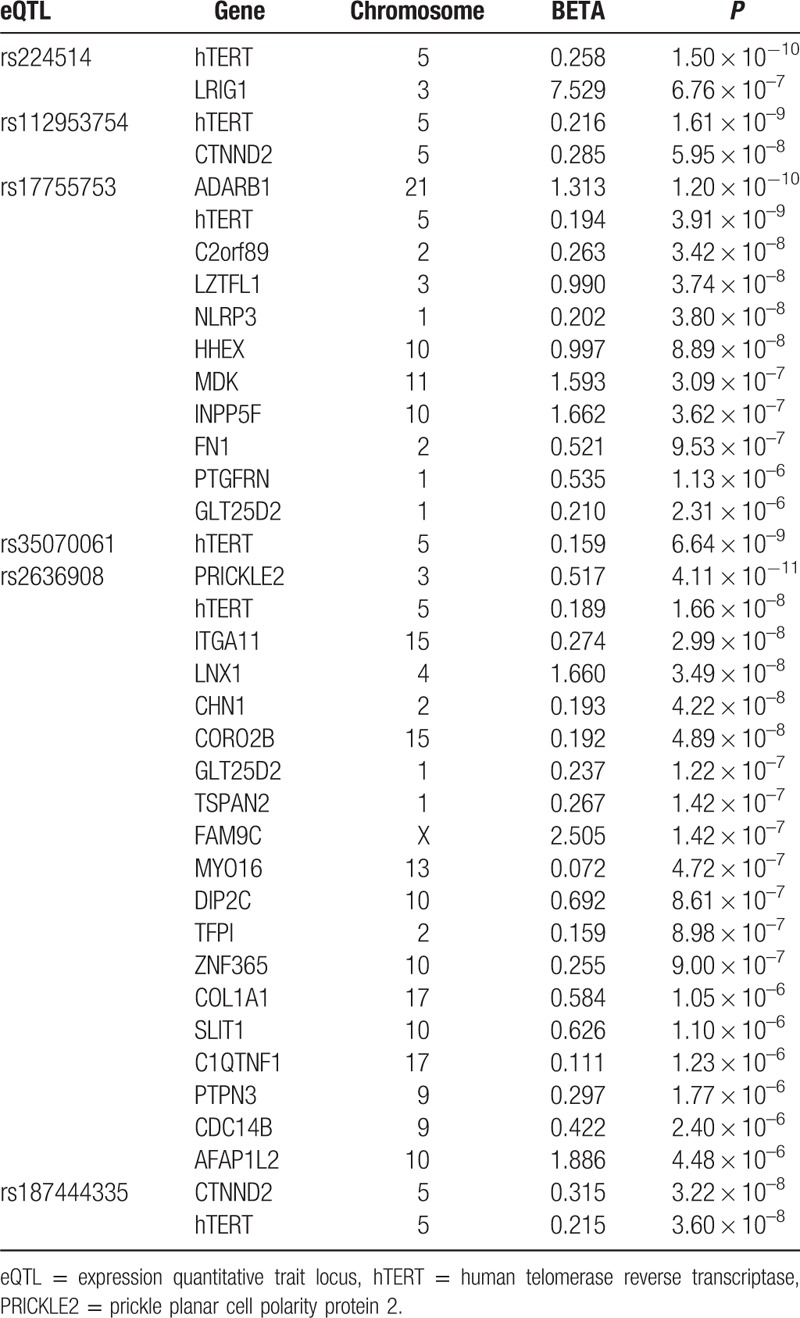
Transcriptome-wide associations of genes with eQTLs identified for hTERT.

## 4 Discussion

The current study identified 6 genetic association signals that might regulate the expression of the gene encoding hTERT and explain partial variability of its expression. In particular, a signal located in the gene of RSPO3 was found. The association of RSPO3 with the expression of hTERT could be supported by previous studies. It has been shown that the R-spondin family can disrupt the inhibition of LRPs caused by DKK1, and thus LRPs activate the Wnt signaling pathway.^[13,14]^ The RSPO3 can enhance angiogenesis and proliferation of human endothelial cells as a Wnt signaling regulator.^[15]^ It has been reported that β-catenin, a critical intracellular signal transducer in the Wnt signaling pathway, can regulate the mRNA expression of hTERT.^[16–18]^ Thus, RSPO3 can induce hTERT through the Wnt signaling pathway.

One of the 6 association signals covered 26 nucleotide variants in strong linkage within LOC105370092. Some of these variants were corresponding to a transcription factor binding sites uncovered by ChIP-Seq (RegulomeDB; Supplementary Table 1), suggesting that these variants might regulate the expression of the uncharacterized ncRNA gene. Significant associations of these variants with the expression of CTNND2 were found (*P* < 4.75 × 10^–6^; Table 2), indicating that the ncRNA might influence the expression of hTERT and CTNND2. The RegulomeDB showed that rs113285167 was found in lymphoblastoid cells as binding sites of transcription factor 12 (TCF12) and CCCTC-binding factor (CTCF) (Supplementary Table 1), which have opposite functions as a transcription factor and a repressor, respectively. The functionally contrasting proteins could bind the locus even in the same cell line (GM12878; ENCODE http://genome.ucsc.edu/ENCODE), but relative binding affinity remained unknown in these studies, especially by alleles. Further research on specific underlying mechanism on their interaction with binding sites is required to understand how gene expression is affected by alleles.

Transcriptome-wide association analysis revealed that the 6 signals associated with hTERT expression were further associated with the expression of many other genes. The most significant association was observed between prickle planar cell polarity protein 2 (PRICKLE2) and rs2636908. Since the PRICKLE2 gene and this SNP were both located in chromosome 3, a regulatory element for expression of PRICKLE2 might include this SNP or other SNPs strongly linked to it. The complex of PRICKLE2 with Vangl2 can regulate the Wnt/planar cell polarity (PCP) pathway that changes cytoskeleton.^[19]^ Genes identified with eQTL of rs2636908 were enriched with cytoskeleton (Supplementary Table 2). Acute withdrawals of TERT in mouse have triggered a rapid change in the expression of genes with the functions in the cytoskeleton,^[20]^ suggesting that hTERT might be involved in cytoskeletal changes associated with the PCP pathway.

The current study dealt with expression of telomerase genes in lymphoblastoid cell lines transformed by Epstein–Barr virus (EBV).^[8]^ The lymphoblastoid cell lines may be suitable for identifying eQTLs of telomerase genes since they demonstrated strong telomerase activity to be immortalized.^[21–23]^ In EBV-infected B cell, expression of ectopic hTERT simultaneously increased with expression of basic leucine zipper ATF-like transcription factor (BATF) which maintains EBV latent status, and hTERT silenced by shRNA can induce B-cell death through transition into lytic cycle of EBV.^[24]^ Thus, lymphoblastoid cells transformed by EBV can be pertinent to hTERT expression studies. Also, we suspect that the Wnt signaling pathway can be instrumental in EBV-transformed lymphoblastoid cells. This is because β-catenins are expressed in lymphoblastoid cells, and moreover, EBV-transformed lymphoblastoid cells increased the expression of β-catenin more than EBV-negative cells.^[25–28]^

Any eQTLs for TERC, another important component of human telomerase, were not found in this study (*P* > 5 × 10^–8^). However, many false negatives are suspected because we employed a conservative significance threshold. Some “suggestive” eQTLs for TERC are presented in Fig. 1 as potential false negatives (10^–5^ > *P* > 5 × 10^–8^).

The current GWAS identified 6 novel eQTLs for hTERT. Some of these eQTLs were involved in the Wnt signaling pathway critical to the production of tumors. Functional studies are needed in order to understand the underlying mechanisms of the Wnt signaling pathway influenced by hTERT. This will provide some evidence to unveil the reason why hTERT expression is associated with tumor.

## Supplementary Material

Supplemental Digital Content

## References

[1] Blackburn EH Epel ES Lin J Human telomere biology: a contributory and interactive factor in aging, disease risks, and protection. *Science* 2015; 350:1193–1198. 26785477 10.1126/science.aab3389

[2] Shay JW Zou Y Hiyama E Telomerase and cancer. *Hum Mol Genet* 2001; 10:677–685. 11257099 10.1093/hmg/10.7.677

[3] Yan P Coindre JM Benhattar J Telomerase activity and human telomerase reverse transcriptase mRNA expression in soft tissue tumors correlation with grade, histology, and proliferative activity. *Cancer Res* 1999; 59:3166–3170. 10397260

[4] Akiyama M Hideshima T Shammas MA Effects of oligonucleotide N3′→ P5′ thio-phosphoramidate (GRN163) targeting telomerase RNA in human multiple myeloma cells. *Cancer Res* 2003; 63:6187–6194. 14559802

[5] Codd V Mangino M van der Harst P Common variants near TERC are associated with mean telomere length. *Nat Genet* 2010; 42:197–199. 20139977 10.1038/ng.532 PMC3773906

[6] Bojesen SE Pooley KA Johnatty SE Multiple independent variants at the TERT locus are associated with telomere length and risks of breast and ovarian cancer. *Nat Genet* 2013; 45:371–384. 23535731 10.1038/ng.2566 PMC3670748

[7] Kote-Jarai Z Saunders EJ Leongamornlert DA Fine-mapping identifies multiple prostate cancer risk loci at 5p15, one of which associates with TERT expression. *Hum Mol Genet* 2013; 22:2520–2528. 23535824 10.1093/hmg/ddt086 PMC3658165

[8] Lappalainen T Sammeth M Friedländer MR Transcriptome and genome sequencing uncovers functional variation in humans. *Nature* 2013; 501:506–511. 24037378 10.1038/nature12531 PMC3918453

[9] Purcell S Neale B Todd-Brown K PLINK: a tool set for whole-genome association and population-based linkage analyses. *Am J Hum Genet* 2007; 81:559–575. 17701901 10.1086/519795 PMC1950838

[10] Barrett JC Fry B Maller JDMJ Haploview: analysis and visualization of LD and haplotype maps. *Bioinformatics* 2005; 21:263–265. 15297300 10.1093/bioinformatics/bth457

[11] Boyle AP Hong EL Hariharan M Annotation of functional variation in personal genomes using RegulomeDB. *Genome Res* 2012; 22:1790–1797. 22955989 10.1101/gr.137323.112 PMC3431494

[12] Huang DW Sherman BT Tan Q DAVID Bioinformatics Resources: expanded annotation database and novel algorithms to better extract biology from large gene lists. *Nucleic Acids Res* 2007; 35:W169–W175. 17576678 10.1093/nar/gkm415 PMC1933169

[13] De Lau WB Snel B Clevers HC The R-spondin protein family. *Genome Biol* 2012; 13:242. 22439850 10.1186/gb-2012-13-3-242 PMC3439965

[14] Aoki M Mieda M Ikeda T R-spondin3 is required for mouse placental development. *Dev Biol* 2007; 301:218–226. 16963017 10.1016/j.ydbio.2006.08.018

[15] Kazanskaya O Ohkawara B Heroult M The Wnt signaling regulator R-spondin 3 promotes angioblast and vascular development. *Development* 2008; 135:3655–3664. 18842812 10.1242/dev.027284

[16] Park JI Venteicher AS Hong JY Telomerase modulates Wnt signalling by association with target gene chromatin. *Nature* 2009; 460:66–72. 19571879 10.1038/nature08137 PMC4349391

[17] Zhang Y Toh L Lau P Human telomerase reverse transcriptase (hTERT) is a novel target of the Wnt/β-catenin pathway in human cancer. *J Biol Chem* 2012; 287:32494–32511. 22854964 10.1074/jbc.M112.368282 PMC3463325

[18] Hoffmeyer K Raggioli A Rudloff S Wnt/β-catenin signaling regulates telomerase in stem cells and cancer cells. *Science* 2012; 336:1549–1554. 22723415 10.1126/science.1218370

[19] Nagaoka T Ohashi R Inutsuka A The Wnt/planar cell polarity pathway component Vangl2 induces synapse formation through direct control of N-cadherin. *Cell Rep* 2014; 6:916–927. 24582966 10.1016/j.celrep.2014.01.044

[20] Choi J Southworth LK Sarin KY TERT promotes epithelial proliferation through transcriptional control of a Myc-and Wnt-related developmental program. *PLoS Genet* 2008; 4:e10. 18208333 10.1371/journal.pgen.0040010 PMC2211538

[21] Sugimoto M Ide T Goto M Reconsideration of senescence, immortalization and telomere maintenance of Epstein–Barr virus-transformed human B-lymphoblastoid cell lines. *Mech Ageing Dev* 1999; 107:51–60. 10197788 10.1016/s0047-6374(98)00131-6

[22] Sugimoto M Tahara H Ide T Steps involved in immortalization and tumorigenesis in human B-lymphoblastoid cell lines transformed by Epstein–Barr virus. *Cancer Res* 2004; 64:3361–3364. 15150084 10.1158/0008-5472.CAN-04-0079

[23] Takahashi T Kawabe T Okazaki Y In vitro establishment of tumorigenic human B-lymphoblastoid cell lines transformed by Epstein–Barr virus. *DNA Cell Biol* 2003; 22:727–735. 14659045 10.1089/104454903770946700

[24] Dolcetti R Giunco S Dal Col J Epstein–Barr virus and telomerase: from cell immortalization to therapy. *Infect Agent Cancer* 2014; 9:8. 24572088 10.1186/1750-9378-9-8 PMC3943417

[25] Shackelford J Maier C Pagano JS Epstein–Barr virus activates β-catenin in type III latently infected B lymphocyte lines: Association with deubiquitinating enzymes. *Proc Natl Acad Sci* 2003; 100:15572–15576. 14663138 10.1073/pnas.2636947100 PMC307609

[26] Morrison JA Klingelhutz AJ Raab-Traub N Epstein-Barr virus latent membrane protein 2A activates β-catenin signaling in epithelial cells. *J Virol* 2003; 77:12276–12284. 14581564 10.1128/JVI.77.22.12276-12284.2003 PMC254275

[27] Morrison JA Raab-Traub N Roles of the ITAM and PY motifs of Epstein–Barr virus latent membrane protein 2A in the inhibition of epithelial cell differentiation and activation of β-catenin signaling. *J Virol* 2005; 79:2375–2382. 15681438 10.1128/JVI.79.4.2375-2382.2005 PMC546559

[28] Everly DN Kusano S Raab-Traub N Accumulation of cytoplasmic β-catenin and nuclear glycogen synthase kinase 3β in Epstein–Barr virus-infected cells. *J Virol* 2004; 78:11648–11655. 15479806 10.1128/JVI.78.21.11648-11655.2004 PMC523297

