# Supplementary material for: Genome-wide identification of expression quantitative trait loci for human telomerase
Source: Medicine (Baltimore). 2016 Oct 21;95(42):e5209. doi: 10.1097/MD.0000000000005209 (PMC5079342; doi:10.1097/MD.0000000000005209)
Supplement: Supplemental Digital Content [file medi-95-e5209-s001.pdf]

Supplementary table 1. Transcription factor binding sites containing nucleotide variants associated with hTERT expression.

SNP	Transcription factor	Cell type
rs117212853	CTCF	A549
	CTCF	Gliobla
	CTCF	GM12865
	CTCF	HCT-116
	CTCF	HeLa-S3
	CTCF	HepG2
	CTCF	HSMM
	CTCF	HSMMtube
	CTCF	K562
	CTCF	MCF-7
	CTCF	NB4
	E2F6	K562
	CTCF	GM12892
	CTCF	H1-hESC
	CTCF	WERI-Rb-1
	RAD21	HepG2
rs113633899	SIN3A	H1-hESC
	TFAP2A	HeLa-S3
	TFAP2C	HeLa-S3
	IKZF1	HSPC
	MYC	MCF-7
rs113285167	SIN3A	H1-hESC
	TFAP2A	HeLa-S3
	TFAP2C	HeLa-S3
	IKZF1	HSPC
	CTCF	GM12891
	CTCF	GM19239
	CTCF	MCF-7
	CTCF	NHEK
	MAX	NB4
	MYC	MCF-7
	TCF12	GM12878
	TCF12	H1-hESC
	CTCF	GM19238
	CTCF	GM12892
rs112953754	CTCF	MCF-7
	IKZF1	HSPC
rs75629604	CREBBP	Jurkat
	E2F6	K562

Supplementary table 2. Functions annotated from enrichment analysis with genes which expressions were associated with rs2636908.

Function	P	Relevant Genes
Linker	0.02	CDC14B, TERT
Cytoskeletal protein	0.03	CORO2B, PRICKLE2, MYO16, LNX1
Actin binding cytoskeletal protein	0.04	CORO2B, PRICKLE2, MYO16
Cell motility	0.04	CORO2B, PRICKLE2, SLIT1
